# Open Health Imaging Foundation Viewer: An Extensible Open-Source Framework for Building Web-Based Imaging Applications to Support Cancer Research

**DOI:** 10.1200/CCI.19.00131

**Published:** 2020-04-23

**Authors:** Erik Ziegler, Trinity Urban, Danny Brown, James Petts, Steve D. Pieper, Rob Lewis, Chris Hafey, Gordon J. Harris

**Affiliations:** ^1^ Open Health Imaging Foundation, Boston, MA; ^2^ Massachusetts General Hospital, Boston, MA; ^3^ Tumor Imaging Metrics Core, Boston, MA; ^4^ Precision Imaging Metrics, Boston, MA

## Abstract

**PURPOSE:**

Zero-footprint Web architecture enables imaging applications to be deployed on premise or in the cloud without requiring installation of custom software on the user’s computer. Benefits include decreased costs and information technology support requirements, as well as improved accessibility across sites. The Open Health Imaging Foundation (OHIF) Viewer is an extensible platform developed to leverage these benefits and address the demand for open-source Web-based imaging applications. The platform can be modified to support site-specific workflows and accommodate evolving research requirements.

**MATERIALS AND METHODS:**

The OHIF Viewer provides basic image review functionality (eg, image manipulation and measurement) as well as advanced visualization (eg, multiplanar reformatting). It is written as a client-only, single-page Web application that can easily be embedded into third-party applications or hosted as a standalone Web site. The platform provides extension points for software developers to include custom tools and adapt the system for their workflows. It is standards compliant and relies on DICOMweb for data exchange and OpenID Connect for authentication, but it can be configured to use any data source or authentication flow. Additionally, the user interface components are provided in a standalone component library so that developers can create custom extensions.

**RESULTS:**

The OHIF Viewer and its underlying components have been widely adopted and integrated into multiple clinical research platforms (e,g Precision Imaging Metrics, XNAT, LabCAS, ISB-CGC) and commercial applications (eg, Osirix). It has also been used to build custom imaging applications (eg, ProstateCancer.ai, Crowds Cure Cancer [presented as a case study]).

**CONCLUSION:**

The OHIF Viewer provides a flexible framework for building applications to support imaging research. Its adoption could reduce redundancies in software development for National Cancer Institute–funded projects, including Informatics Technology for Cancer Research and the Quantitative Imaging Network.

## INTRODUCTION

Oncology imaging informatics encompasses a vast array of workflows, tools, and features that end users have come to rely on to support their research and clinical trial needs. However, many applications currently use locally installed desktop software as so-called thick or thin imaging clients. Although feature-rich desktop imaging applications address many oncology needs, desktop-installed tools also have significant limitations. Locally installed software tools are often limited to specific operating systems and can be challenging to remotely access and update when installed inside hospital environments. Additionally, installing, supporting, and troubleshooting desktop applications may be time consuming and require local information technology (IT) desktop support, or in some cases, installation may not be permitted on institutional computers.

Furthermore, cloud computing is quickly becoming an attractive computing model for biomedical research.^1^ Compared with standard desktop workstations and local high-performance clusters, public cloud providers offer pay-as-you-go, on-demand, configurable resources. They can provide a secure and cost-effective infrastructure that is both highly available and scalable. Hospitals and research sponsors are more often turning to cloud computing to facilitate the large-scale data analyses that are now common in research environments. As more data are relocated to cloud resources, Web-based imaging applications become the most practical approach to interacting with remote data sources. Zero-footprint Web viewers enable display and manipulation of images within a Web browser, with no additional software installed on the user’s local computer. Because patient data often exist in silos at individual sites, applications that use cloud storage can simplify data sharing across sites, ease collaboration, and improve patient care.

**CONTEXT**

**Key Objective**
To develop a user-friendly Web-based medical image viewer framework that can be adapted to satisfy the needs of cancer researchers and clinicians.
**Knowledge Generated**
The viewer has been extended and adopted by multiple clinical research platforms (eg, Precision Imaging Metrics, XNAT, LabCAS, ISB Cancer Genomics Cloud) and commercial groups. It has also been used to build custom imaging applications for training and education purposes (eg, eContour, Crowds Cure Cancer).
**Relevance**
Clinicians can use the framework to develop purpose-built applications for small subsets of patients, experiment with new imaging tools, or produce training modules. The framework will support the transition to cloud computing and data storage in research environments, which will improve patient data accessibility across sites.

Although many commercial radiology vendors have begun adopting Web-based imaging applications, they often fall short of the needs of cancer imaging researchers and clinicians. A common desire is the flexibility and extensibility to integrate custom tools and user interfaces to support bespoke research workflows and purpose-built applications for small subsets of patients. In these cases, researchers often turn to open-source options to develop their own plugins or software packages. At present, however, there is an unmet need for well-supported, extensible, professional-grade, user-friendly, and interoperable Web viewer technology.

The Open Health Imaging Foundation (OHIF) Viewer platform is being developed to fulfill this need. The extensibility of this zero-footprint Web-based platform enables customization and integration with other applications. Additionally, its components can be reused to rapidly develop new applications and will help enable the transition to cloud storage and computing for cancer research tools. The OHIF framework has been used to build numerous research systems, as well as clinical applications that have obtained US Food and Drug Administration 510(k) clearance and are available for use in clinical practice. Its libraries have been used to build training applications for radiation oncologists, as well as tools for measurement of morphomic markers and an online objective structured clinical examination application for radiology education.^2-4^

In this article, we will discuss the current architecture of the OHIF Viewer and provide a basic overview of its functionality and how its components have been integrated into various applications over the past few years. Finally, we will discuss future plans for the project and how they will affect the cancer research community.

## MATERIALS AND METHODS

**FIG 1.**
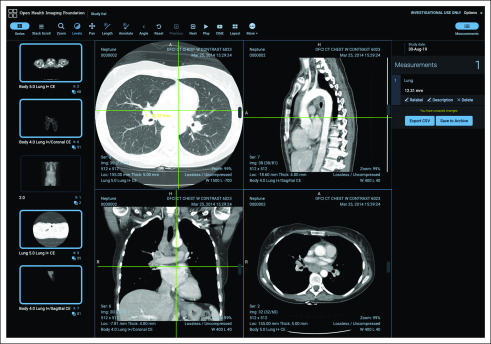
Open Health Imaging Foundation Viewer user interface: an overview of the viewer user interface showing the toolbar, sidebar panels, and viewports.

The OHIF Viewer (Fig 1) is a single-page Web application that can function on any modern Web browser (ie, Chrome, Firefox, Internet Explorer ≥ 11). The application supports connectivity to image archives over industry-standard Web services (eg, DICOMweb^5^), as well as custom data sources, including data from the local file system. Architecturally, it is split into several libraries, which each contribute to the overall viewer functionality. The repository includes a user interface component library made up of React^6^ components, which can be used independently to build custom user interfaces. Business logic and internationalization files are encapsulated in independent reusable packages. These packages are automatically published on NPM^7^ using semantic versioning guidelines. Unit and end-to-end tests are run on a continuous integration service, and branch-specific preview versions (ie, deploy previews) are produced to simplify manual testing. These features allow the platform to be updated frequently while ensuring stability.

Cornerstone^8^ is used by default for image retrieval, decoding, and rendering of Digital Imaging and Communications in Medicine (DICOM) images. It can leverage CPU- and GPU-based rendering to display medical imaging data sets. A pluggable image loader framework provides support for DICOM instances and retrieval of individual image frames, which is critical for performance when viewing large multiframe images (eg, for breast tomosynthesis). All DICOM transfer syntaxes are supported, and images are decoded inside background threads to ensure that the user interface continues to function fluidly. Cornerstone Tools provides a framework for developing measurement and annotation tools, which can be used for image analysis on desktop and touch devices. It includes tools such as interactive window/leveling, measurements such as length and angle, region-of-interest measurements (eg, rectangle, ellipse), segmentation brush and scissors tools, a magnifying glass, and CINE tools, among others. Creation of DICOM objects is available through dcmjs,^9^ which allows us to easily create DICOM structured reports, secondary captures, and segmentations within the Web browser. These can be downloaded to the user’s desktop or pushed directly to picture archiving and communication system (PACS).

**FIG 2.**
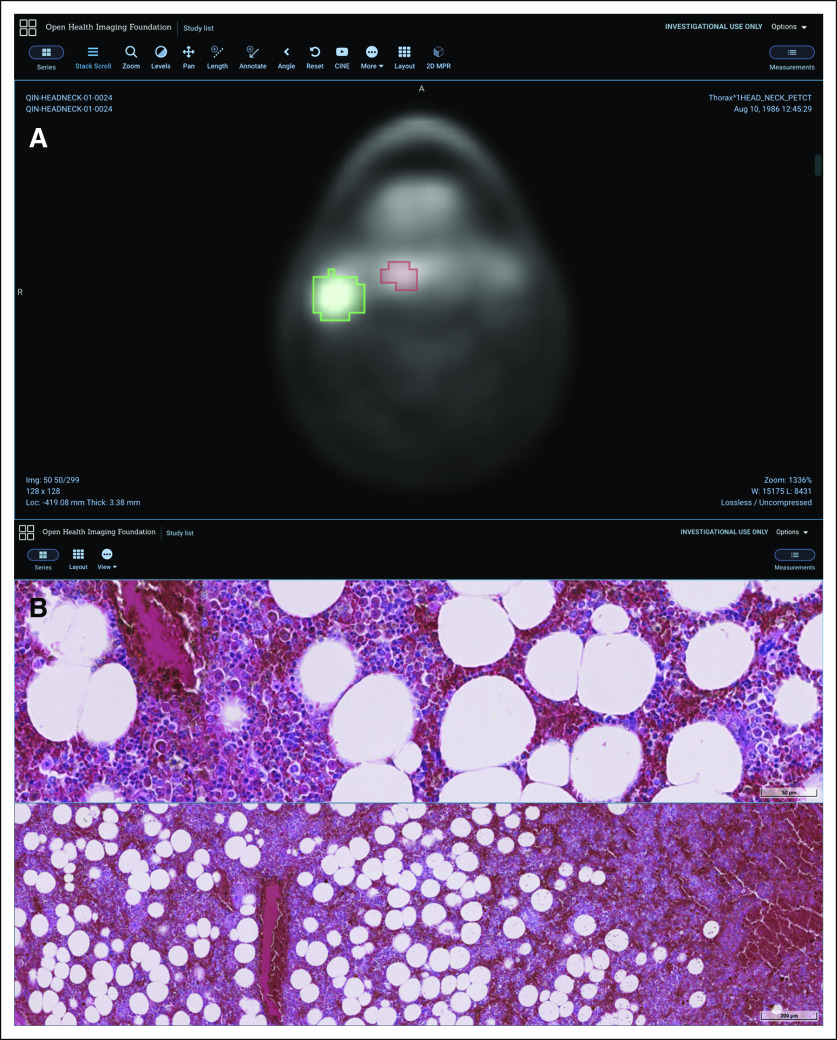
Open Health Imaging Foundation (OHIF) Viewer functionality. (A) Visualization of a DICOM segmentation file showing lesions segmented on a fluorodeoxyglucose positron emission tomography image in a patient with head and neck cancer. Data from the QIN-HEADNECK data set.^36-38^ (B) Pathology whole-slide image visualization inside OHIF using the DICOM microscopy extension. Data from DICOM NEMA Working Group 26 Public Datasets.^39^

The framework provides an application programming interface (API) to allow extensions to provide alternative data display pathways. This can be useful for specific use cases, such as three-dimensional (3D) image fusion, where using a 3D-focused rendering library is desirable. For this functionality, OHIF leverages VTK.js,^10^ a JavaScript library for scientific visualization that is the Web successor to the ubiquitous Visualization Toolkit C++ library.^11^ An extension that provides VTK.js support for advanced visualization, such as multiplanar reformatting, volume rendering, maximum intensity projection, and rendering surface models, is currently available in OHIF and under continuing development. Training materials for OHIF with VTK.js have been made freely available to encourage developer adoption.^12^ Additionally, an extension to support whole-slide microscopy images using DICOMweb is available^13^ (Fig 2). A core goal of the viewer architecture is extensibility, because this will reduce redundant work across development groups. The platform supports a number of different extension points, as summarized in Table 1.

**TABLE 1.**
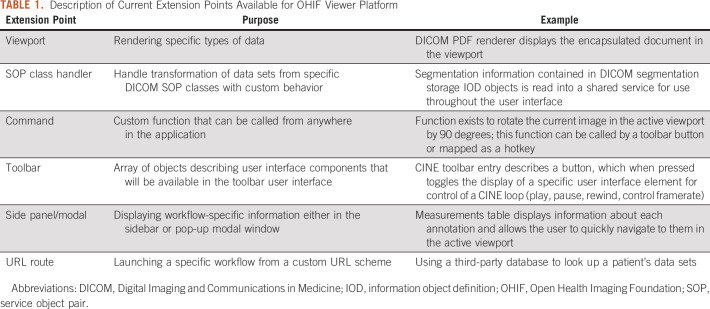
Description of Current Extension Points Available for OHIF Viewer Platform

Simple, secure, cost-effective deployment is a key focus of the OHIF Viewer development. The viewer can be hosted using any static-site hosting solution or deployed via Docker. To configure the viewer, the developer only needs to specify a DICOMweb server and an optional identity provider application for user authentication. OHIF supports the widely available OpenID Connect authentication protocol, as well as custom authentication methods, to ensure secure communication to Web services, such as when retrieving data or storing results back to PACS. Support for DICOM storage using the Google Cloud Healthcare API is also available, which offers a secure, scalable backend without the need to manage a server.

The OHIF Viewer and all core supporting dependencies (ie, Cornerstone, dcmjs) are available open source under a commercially permissive MIT License, which we believe has allowed the codebase to reach a wide audience. Software development is managed on GitHub for OHIF,^14^ Cornerstone,^15^ and dcmjs.^9^ Additional support is available through a Google Groups forum.^16^

Initially, the OHIF Viewer was released as a reference application to demonstrate basic image review features using the OHIF framework.^17^ Alongside the OHIF Viewer, the LesionTracker, an oncology response assessment application, and the OHIF Standalone Viewer, a client-only version of the basic viewer, were maintained. It became challenging to support and maintain three viewers and clear that the wider community was most interested in the client-only viewer above the two client-server systems. At the end of 2018, the core OHIF team decided to make two major changes to our approach: first, we would merge core LesionTracker functionality to allow us to maintain only a single viewer, and second, we would rewrite the application as a client-only viewer, indifferent to the back-end with which it is connected. These changes allow us to more effectively manage project and community requirements, making support and ongoing development more sustainable.

## RESULTS

Since the founding of OHIF in 2015, we have witnessed widespread adoption of the OHIF Viewer and its underlying components in various use cases. There have generally been three approaches for using the application: used as a generic radiology viewer, modified to create an extended version of the viewer application, and used piece by piece to build custom imaging applications.

The viewer provides a simple avenue for white labeling (ie, replacing the OHIF logo) and theming. This functionality has been leveraged to integrate it as a radiology viewer for numerous projects, such as OsiriX, a commercial desktop imaging application that also provides a Web portal for the user’s cloud-accessible data, the Early Detection Research Network’s LabCAS (Laboratory Catalog and Archive Service), a partnership between NASA and the National Cancer Institute (NCI),^18^ the open-source Kheops^19^ image sharing platform from the OxiriX Foundation,^20^ the Institute for Systems Biology’s Cancer Genomics Cloud,^21^ and The Cancer Imaging Archive (TCIA) Web site. In addition, it will form the foundation for the upcoming NCI Imaging Data Commons^22^ and for radiology image viewing in the National Heart, Lung, and Blood Institute’s BioData Catalyst project.^23^ These integrations were facilitated by our focus on standards-based interfaces, which allow OHIF to easily communicate with third-party components.

**FIG 3.**
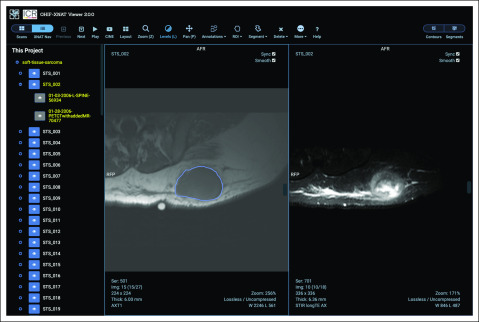
Open Health Imaging Foundation (OHIF)–XNAT user interface: a customized version of the OHIF Viewer has been integrated into the XNAT imaging informatics platform. Users can navigate through their XNAT projects and patients and save and restore measurements, contours, and segmentation label maps to the XNAT database. Reproduced with permission.

**FIG 4.**
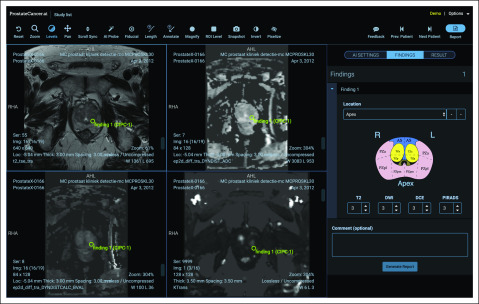
ProstateCancer.ai user interface. Training and artificial intelligence (AI) demonstration application built on the Tesseract-MI platform. This application demonstrates how to perform Prostate Imaging-Reporting and Data System reporting and includes an AI probe tool to estimate whether the current position contains a clinically significant prostate cancer finding. The sidebar panel includes a clickable map of the prostate to facilitate results reporting.

Several platforms have modified the OHIF Viewer for their specific use cases. One example is XNAT, an informatics platform that supports imaging research. In the XNAT case, a customized (ie, forked) version of the viewer is being maintained, which adds deeper integration with the XNAT project hierarchy as well as support for radiotherapy and segmentation workflows^24^ (Fig 3), although XNAT is planning to merge with the main source code repository going forward. Another instance is Tesseract-MI, an open-source, Web-based platform for deployment of artificial intelligence models^25^ (Fig 4). Tesseract’s initial demonstration application provided Prostate Imaging-Reporting and Data System training and reporting functionalities.

**FIG 5.**
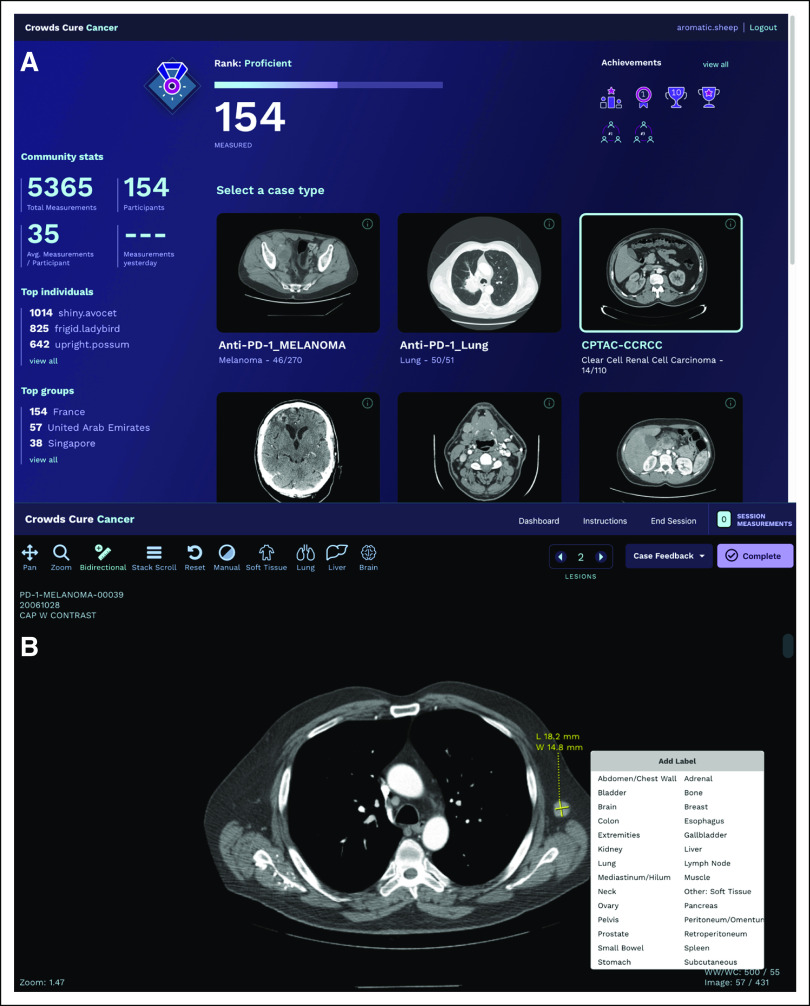
Crowds Cure Cancer user interface. (A) Dashboard showing the user’s current rank, number of measurements completed, and achievement badges earned. From this section, a data collection can be opened for measurement. (B) The viewer interface for the application, showing a lesion with the lesion-labeling user interface. The application is built to allow users to make lesion measurements efficiently to crowd source ground-truth data for publicly available cancer imaging data.

The modularity of the components has made it easy to build new custom applications. One such example is Crowds Cure Cancer,^26^ an application for crowdsourcing radiology image annotations (Fig 5). This application was developed to enhance the value of publicly available data sets from TCIA, because many were provided to the repository without annotations of diseased tissue. Roughly 2,700 cases were incorporated from 18 different TCIA collections and stored in an open-source DICOM archive (dcm4chee).^27^ Because common functions such as user authentication, study metadata retrieval via DICOMweb, image decoding, rendering, and annotation tools could be easily imported from OHIF, the majority of the effort needed to develop the application was spent on the workflow, user interface, and application-specific features such as storing serialized annotations into a custom database and aggregating these to provide a participant leaderboard. The application was built with a responsive design so that it could be used on mobile phones, tablets, and desktop computers and leveraged the touch support for image interaction and annotation provided by Cornerstone. Participants were asked to perform bidirectional measurements of all metastatic disease in a set of computed tomography studies, as well as to provide location labels for their measurements. At the 2018 Radiologic Society of North America (RSNA) conference, > 5,000 measurements were collected in < 1 week. The data collected at the 2017 and 2018 RSNA annual meetings are available on TCIA in both DICOM structured reports and CSV files.^28,29^ Using the OHIF framework components, this crowdsourcing application could be modified in the future to use alternative measurement types, such as segmentation tools.

Another example is Precision Imaging Metrics (PIM), a cloud-hosted clinical trial imaging informatics system developed by the Tumor Imaging Metrics Core of the Dana-Farber/Harvard Cancer Center, which includes OHIF-based image viewing and measurement tools. The PIM Web viewer is in the process of being deployed at eight NCI-designated cancer centers nationwide. In addition, the Center for Clinical Data Science at Massachusetts General Hospital and Brigham and Women’s Hospital used OHIF components to develop a custom image annotation application to gather training data for machine learning. The National Institutes of Health TB Portals repository, a platform for data sharing and analysis to combat drug-resistant tuberculosis, is also leveraging OHIF platform components.^30^

According to GitHub, there are 215 unique repositories using Cornerstone as a dependency as of January 2020. On the OHIF homepage, we have been distributing a prototype Windows-only desktop-installable version of the viewer, which has been downloaded roughly 8,600 times since May 2018. Through GitHub’s API, we have identified 136 unique code contributors to the OHIF Viewer and Cornerstone libraries since their inception. In July 2019, we began efforts to crowdsource translations for the user interface component library. Since then, community members have provided comprehensive translations for Chinese, Japanese, Vietnamese, Brazilian Portuguese, and Spanish.

## DISCUSSION

It is clear that the demand for Web-based medical imaging applications in clinical oncology is growing significantly. This shift is being pushed by the complexities around deployment and maintenance of desktop applications and the growing acceptance of cloud computing at hospitals. Many groups invest in the development of custom imaging solutions to support their specific cancer research objectives yet rely on similar core functionality inherent to most imaging-based applications. Research teams often spend substantial resources developing basic data ingestion, management, and visualization functionality rather than validating and hardening their custom tools. Open-source imaging libraries and viewers can reduce this duplication of effort. Under permissive software licenses, open-source tools can achieve broad adoption. The OHIF framework has become widely implemented in Web-based medical imaging applications because of its quality, support, ease of use, and integration.

There are a number of alternative quantitative imaging platforms available that focus on oncology workflows. The most similar in nature to the OHIF Viewer is ePAD,^31^ which is open source and developed at Stanford Radiology. ePAD aims to be an entire system for quantitative imaging; it is distributed with a PACS, a measurement database, and a Web services server. This comprehensive setup allows users to easily get started with an entire quantitative image analysis platform but makes it more challenging for developers to integrate the individual components into other systems. Additionally, it currently relies on Google Web Toolkit, a legacy Java framework no longer being actively developed by Google. Another alternative is the Web-based response assessment system developed by Yang et al^32^ at Columbia University. The core drawback of this system is that it is not open source, despite being based on Weasis,^33^ a mature free open-source image viewer. Furthermore, although it can be launched with remote imaging studies, it requires installation on the workstation and is not in fact a zero-footprint application.

In quantitative imaging, it remains a challenge to share new tools with the community. An aim for OHIF is to become a platform for distribution of novel tools. If the viewer is flexible enough to be widely integrated, extensions that are developed can be more easily translated from in-house use to validated clinical research software, which can be used by the larger cancer center community. An existing extensible research platform is 3D Slicer,^34^ which has been extraordinarily successful and adopted worldwide for imaging research. Its key drawbacks are that it is a desktop imaging application that requires local installation, and it is so feature rich that it can be challenging to use by clinicians.

Future plans for the OHIF Viewer are focused on advanced visualization, improved support for developers, and development of an avenue for cancer researchers to use the software without IT support. In recent years, the use of machine learning in radiology has skyrocketed. As a consequence, the most commonly requested feature for OHIF has been robust configurable segmentation tools to allow clinicians and researchers to review and correct machine-generated label maps (Fig 2). Developing this feature into an optimized workflow for the Web is one of the top priorities for the future. Similarly, data storage of annotations in DICOM structured reports will allow researchers to more easily curate data sets.^35^

To ensure the longevity of the OHIF platform, it is essential to increase community awareness and reduce barriers for both developers and end users. As adoption has grown, it has become challenging for our team to support the community to solve imaging informatics issues in addition to those related to deployment and security for Web applications. We intend to broaden our documentation on these issues and produce training videos to help avoid confusion. We aim to build an extension development guide including usability guidelines to help plugin authors build user-friendly imaging tools. One critique of the OHIF project is that adoption is challenging for cancer researchers on their own. Most users of OHIF are using it as part of another product. Without an IT professional, it is challenging for clinicians to set up a secure system with the OHIF Viewer to evaluate their own project data, because they need to set up an image archive and authentication system as well. A key issue for future work will be to identify a simple local deployment approach for nontechnical end users.

We believe that OHIF has the potential to become ubiquitous as a clinical oncology imaging viewer. With the development of extensions and guidelines for custom workflows, this platform will be able to facilitate cross–cancer center collaboration by simplifying access to data stored in the cloud, aligning development resources, and providing a mechanism to support tool sharing through a clinician-friendly user interface.
